# Numerical simulation and experimental research on mechanical behaviour of hydraulic disc brakes based on multi-body dynamics

**DOI:** 10.1038/s41598-022-21960-4

**Published:** 2022-11-03

**Authors:** Wenyue Zhang, Cunbao Zhao, Pengyu Chen, Enli Chen, Tianyu Lei

**Affiliations:** State Key Laboratory of Mechanical Behaviour and System Safety of Traffic Engineering Structures, Shijiazhuang Tiedao University, Shijiazhuang, 050043 China

**Keywords:** Engineering, Mechanical engineering

## Abstract

As a vital road construction machine, the bridge erecting machine works in a very complex environment. The brake as an important link to maintain the safety and stability of the bridge erecting machine, it will have serious consequences if it is failed. Establishing the brake simulation model and specifying the fault characteristics according to the actual operation status will make it more efficient to find the cause of faults and maintain the safety of machine for a long time. A simulation model of brake of the bridge erecting machine was established by Adams. The brake arms and center axis with obvious data characteristics were flexibly processed, and finite elements were analysed using Abaqus. To verify the accuracy of simulation models, rectangular rosettes were applied to special geometric points, and the strain data were gathered by using the DH3816N collector and compared with the simulation model. The Adams kinematic simulation model was used to simulate the actual operating conditions by the experiment of the disc springs. Two typical fault phenomena were selected: reduced insufficient of disc spring and oil pressure, and two fault characteristics were extracted: variation of the brake shoe clearance and variation of the braking system pressure. When the brake generates the fault characteristics, the normal operation of the brake will not be affected if the fault characteristics are in the first stage. When the fault characteristics are beyond the critical threshold, the faults phenomena of the brake are generated. The results of the simulation experiments proved that the method of using the simulation model to extract the fault characteristics of the braking system and distinguish the causes of the fault was feasible.

## Introduction

Reportedly, the Chinese special equipment accident rate remains high, which is still 5–6 times higher than in industrialized countries. The situation is still grim^1^. With the construction of the Chinese road traffic network, bridge erecting machines have been widely used in bridge engineering due to their high efficiency. The braking system is an essential part of the safety performance of a bridge erecting machine. In 1995, during the construction of the Huangqi Bridge on the Southwest Ring Expressway in Guangzhou, a GT130 guide-beam bridge erecting machine suffered a severe accident due to rain erosion of the winch^2^. Therefore, it is of great importance to perform simulation experiments on the braking system to extract fault characteristics and monitor the condition of the system.

In recent years, with the gradual maturity and development of railway transportation construction, more and more attention has been paid to the study of bridge erecting machines. For a bridge erecting machine, the braking system is an important part of its structure, which has an important influence on its safety performance. The detection and determination of the status of the bridge erecting machine mainly rely on workers for judgment at the construction site. But it is expensive, time-consuming and cannot detect real-time status. At present, many researchers have carried out investigations of the safety state of the bridge erecting machine structure, but few studies have established a simulation model for the braking system to analyse the working condition of the system. Cheng Y^3^ analysed the type of fault and diagnosis methods of the bridge erecting machine for the detection of faults of bridge erecting machines on-site. It proposed to use displacement deflectometer to obtain the curvature of deflection influence. This method was applied to the main structure of the bridge erecting machine connection assembly for fault diagnosis. However, it cannot provide effective monitoring and analysis for braking system faults. Li X^4^ established a fault tree model based on the overturning accident of bridge erecting machines for analysis, classified the fault characteristics according to different fault causes. Finally, it divided the overturning accident causes into manufacturing defects, personnel operation errors and management negligence. It put forward a corresponding management and control measures. Chen N^5^ established a multi-sensor bridge erecting machine safety monitoring system based on the security monitoring of the large machinery and equipment and carried out the system feature classification of the different working conditions of the bridge erecting machines. Finally, the validity order of safety monitoring information of the multi-sensor sampling points were gained, which can direct optimal scheduling of the position of sensors in large-scale mechanical equipment systems. After that, Chen^6^presented a new discretization algorithm with continuous attribute based on the simplified PCNN (Pulse Coupled Neural Network) model. Measured signals of the bridge erecting machine safety monitoring system are computed on this algorithm, which provided an important reference for establishing the safety monitoring platform for the braking system of the bridge erecting machine. Zhang^7^analysed the behaviour characteristics of the steel wire rope during the walking process of the bridge erecting machine, and tested the damping coefficient of the steel wire rope under the action of beams with different lengths, which provided a reference for the force analysis of the braking system of the bridge erecting machine in the working condition. Zhang C^8^ used ANSYS for finite element modeling simulation. However, the method is only for structural fault analysis. The accuracy of the simulation model was not verified by being tested. LÜ W^9^ described each link for the fault detection of in-service box girder bridge erecting machines on-site. Whereas, they did not establish the simulation model or simulations of fault for the braking system of the bridge erecting machine. The fault characteristics could not be clarified. Zhou X^10^ constructed a fault tree model of the basic causes of faults of braking system based on logical analysis and used binary decision diagrams for analysis. The references for the analysis of the causes of faults of brakes had been provided. For the brakes of large equipment such as vehicles and mine hoists, the simulation model is a very feasible method for analysis, which is worthy of reference^11–14^. In addition, there are several studies that provided the basis for using experiments to verify the feasibility of simulation models^15–17^.

**Figure 1:**
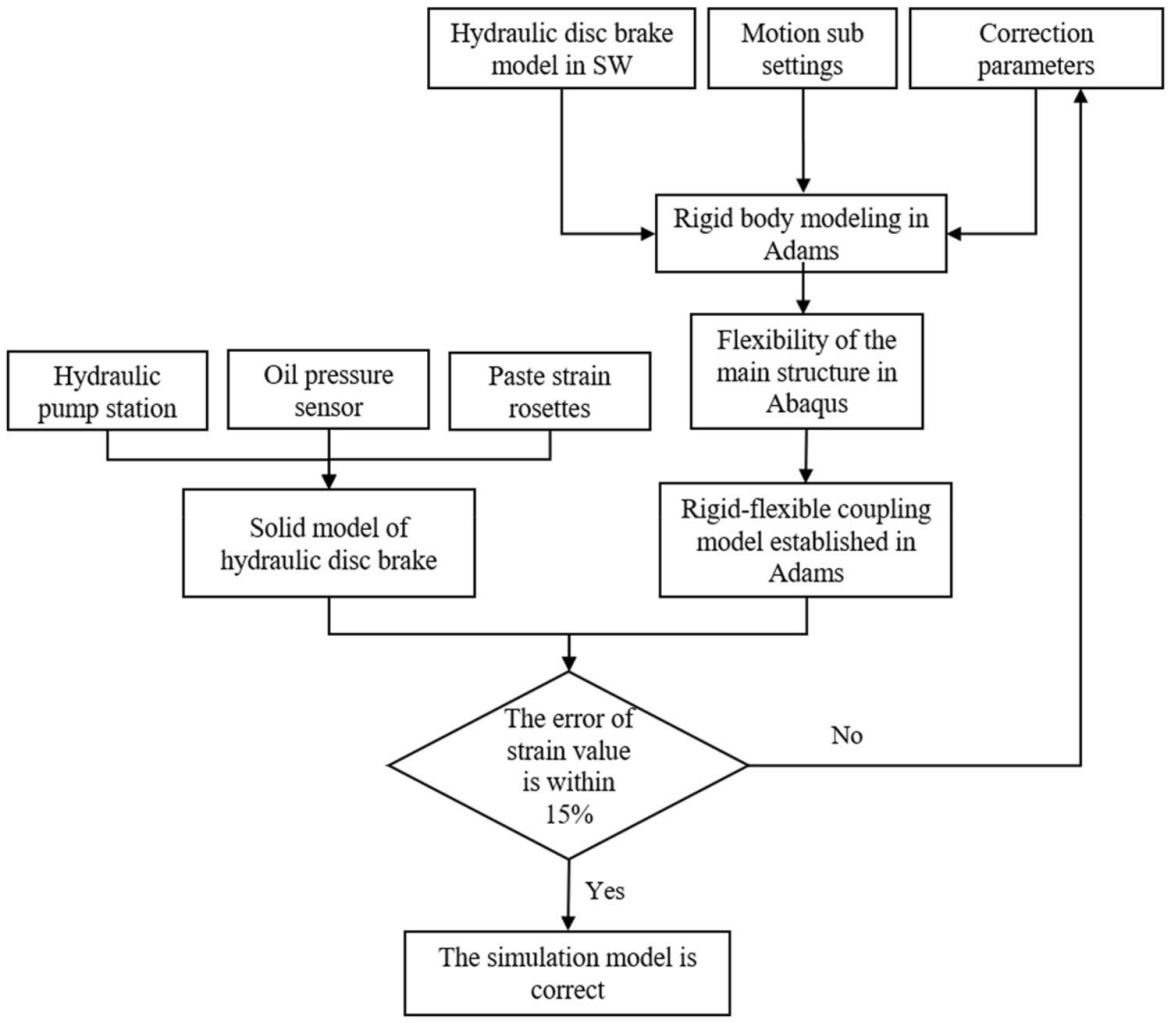
The analysis process of simulation and experiment.

To sum up, this paper innovatively uses the method of establishing a simulation model and experimental verification to extract and determine the faults of the brakes of bridge erecting machine, which greatly changes the monitoring and maintenance methods of such large-scale mechanical equipment, reducing maintenance costs and the waste of resources. It fills the research gap for the brakes of the bridge erecting machine.To a certain extent, it promotes establishing a real-time monitoring platform for large-scale equipment, which greatly maintains the safety and stability state of large-scale mechanical equipment. Figure 1 shows the analysis process of simulation and experiment.

## Modeling the rigid-flexible coupling of brakes

Multi-body dynamics is based on classical mechanics and is divided into multi-rigid-body system dynamics, multi-flexible-body system dynamics, and rigid-flexible coupled system dynamics according to different research objects. Simplifying each component in the system into a rigid body is a research method of multi-rigid body dynamics. The model is established according to the multi-rigid body dynamics which ignores the flexibility characteristics of the component, and the strain analysis of components cannot be carried out using this kind of model. In contrast, the main feature of multi-flexible body system dynamics is the simultaneous occurrence of rigid body motion, deformation motion and the mutual coupling between the two motions. In the simulation process of the brake, the brake arm and the central axis generate stress and strain under the joint action of the disc spring and the hydraulic cylinder which have noticeable data characteristics. The accuracy of model is proved by comparing with the test data of the main structure. In order to simplify the calculation process, it is necessary to transform the bilateral brake arms and the central axis of the brake into a flexible body and establishes the rigid-flexible coupling model of the brake.

When the multi-body dynamics equations of rigid-flexible coupled system is constructed, the system can be divided into two parts: a rigid body and a flexible body. Each of the parts can establish the separate equation according to the theory of multi-body dynamics for a rigid body. The flexible equations are established by the finite element method. The hinge-cut method is used to generate a derivation tree from a closed-loop system consisting of N objects. This system includes deformed body ${\text{B}}_{{\text{i}}}$, which consists of discrete $l$ nodes. Assuming ${\text{e}}^{{\text{r}}}$ is the absolute coordinate system, then the centroid ${\text{C}}_{{\text{i}}}$ of ${\text{B}}_{{\text{i}}}$ is at the coordinate of ${\text{e}}^{{\text{b}}}$ before the deformation. This coordinate can make limited movement and sliding relative to ${\text{e}}^{{\text{r}}}$. The mass of the ${\text{P}}\left( {{\text{P}} = 1, \ldots ,{\text{l}}} \right)$ node is ${\text{m}}_{{\text{P}}}^{^{\prime}}$, the absolute vector diameter of node P is ${\text{r}}_{{\text{i}}}^{{\text{p}}}$, and the translation vector is ${\text{u}}_{{\text{i}}}^{{\text{p}}}$. The radius vector relative to the centroid ${\text{C}}_{{\text{i}}}$ is ${\text{S}}_{{\text{i}}}^{{\text{p}}}$, the radius vector is ${\text{S}}_{{{\text{io}}}}^{{\text{p}}}$ when it is not deformed, and the absolute radius vector of centroid ${\text{C}}_{{\text{i}}}$ is ${\text{r}}_{{\text{i}}}$^18^. The deformation of a node in modal coordinates is represented by:

1 $$u_{i}^{p} = {\Phi }_{{\text{i}}}^{{\text{p}}} a_{i}$$

where ${\Phi }_{{\text{i}}}^{{\text{p}}}$ is the modal vector array of P-nodes after translation, and $a_{i}$ is the modal coordinate matrix of the deformer $B_{i}$.

Based on the above equations, the s-th modal vector can be calculated.

2 $$\Phi _{i}^{p} = \left( {\varPhi _{1} \ldots \varPhi _{s} } \right),a = \left( {a_{1} \ldots a_{s} } \right)^{T}$$

**Figure 2:**
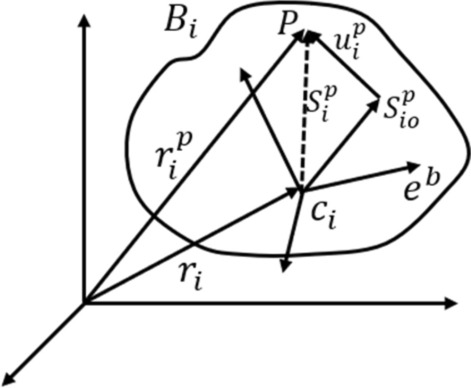
Single deformation body representation.

As shown in Fig. 2, the node deformation can be represented by its relative vector as

3 $$S_{i}^{p} = S_{io}^{p} + u_{i}^{p}$$

The vector relationship between the absolute and relative radius vector of node k is as follows

4 $$r_{i}^{p} = r_{i} + S_{i}^{p}$$

Based on the absolute coordinate system $e$, it assumes that its angular velocity vector parameter is $\omega$, so that the acceleration of the node based on the absolute coordinate system $e$ can be expressed as.

5 $$\overline{r}_{i}^{p} = B_{i}^{p} \upsilon ,\ddot{r}_{i}^{p} = B_{i}^{p} \dot{\upsilon } + \omega^{p}$$

6 $$B_{i}^{p} = \left( {I_{3} - \tilde{\rho }^{{p\varPhi^{p} }} } \right),\omega_{i}^{p} = 2\overline{\omega }\varPhi^{p} \overline{a} + \overline{\omega }^{2} \rho^{p}$$

where $\upsilon$ -the generalized velocity of the object in absolute coordinates, $\upsilon = \left( {\overline{r}^{T} \omega^{T} \overline{a}^{T} } \right)^{T}$.

According to the Jourdain's principle, the dynamic equation of $b_{i}$ is as follows:

7 $$\delta \upsilon^{T} \left( { - M\overline{\upsilon } + f} \right) = 0$$

The kinetic equations of the system can be expressed as

8 $$\mathop \sum \limits_{i = 1}^{N} \delta \upsilon^{T} \left( { - M\overline{\upsilon } + f} \right) + \delta W = 0$$

Applying the single direction recursive construction method, the following recursive relation is obtained^19^.

9 $$\upsilon_{i} = G_{io} \upsilon_{o} + \mathop \sum \limits_{{p:B_{p} \le B_{i} }} G_{ip} \dot{y}_{p}$$

10 $$\dot{\upsilon }_{i} = G_{io} \dot{\upsilon }_{i} + \mathop \sum \limits_{{p:B_{p} \le B_{i} }} \left( {G_{ip} \ddot{y}_{p} + g_{ip} } \right)_{i,p = 1, \ldots ,N}$$

$$y_{i} = \left[ {\begin{array}{*{20}c} {q_{i}^{T} } & {a_{i}^{T} } \\ \end{array} } \right]$$ ,where $q_{i}$ is the hinge-relative coordinate. $a_{i}$ is the modal coordinate matrix of the flexible body, and $y_{i}$ is the position-attitude coordinate array of $B_{i}$. The rigid-flexible coupling term is covered in $f_{i}$. Let $y = \left[ {y_{1}^{T} \ldots y_{N}^{T} } \right]^{T}$. It represents the position-attitude coordinate array of the multi-flexible body system . The multi-body dynamics equation of the derivation tree system is derived in the modal coordinate and hinge-relative coordinate as follows

11 $$Z\ddot{y} = z$$

where $Z$ is the quality matrix for derivation tree systems and $z$ is the force matrix of the derivation tree system.

According to the classical mechanics theory, the constraint equation of multi-body dynamics for constructing the closed-loop system of a derivation tree is as follows:

12 $$\Theta \; = \;0$$

According to the multi-body dynamics theory, the multi-body dynamics equation for constructing a closed-loop system of derivation tree is as follows:

13 $$\left\{ {\begin{array}{*{20}c} {Z\ddot{y} + {\uptheta }_{\mu }^{T} = z} \\ {{\uptheta }_{y} \ddot{y} = \zeta } \\ \end{array} } \right.$$

where ${\uptheta }_{y}$ is the Jacobi matrix of the constraint equation for this system, $\mu$ is the Lagrange multiplier for the system, and $\zeta$ is the right-hand in the acceleration part of the constraint equation for this system.

The above formula is the equation system of the multi-body dynamic system, which is a differential–algebraic equation system, and its holomorphic quadratic differential is as follows:

14 $$Z\ddot{y} = z,z^{*} = z - {\uptheta }_{y}^{T} \left( {{\uptheta }_{y} Z^{ - 1} {\uptheta }_{y}^{T} } \right)^{ - 1} \left( {{\uptheta }_{y} Z^{ - 1} - \zeta } \right)$$

The multi-body dynamics equation is established through the flexible body modal and hinge-relative coordinate is shown in (14). There are coupling terms in the generalized mass matrix in this equation. This dynamic model includes rigid-flexible coupling effect, which can simultaneously solve the system's large-scale rigid body motion and elastic deformation. This is the fundamental difference between the dynamics of flexible multi-body systems and previous dynamics of flexible structures.

The hydraulic disc brake structure consists of a brake arm, a center axis, a disc spring set, and a hydraulic cylinder. The model simplifies the calculation process by reducing a disc spring set to a spring.

First, a multi-body dynamics model was created in Adams. The accuracy of its model parameters largely influenced the accuracy of the dynamic simulation analysis of brakes. It is essential that the parameters used in the modeling are as close to the actual values as possible^20^.

There are four parameters used in modeling: geometric parameters, mass parameters, mechanical parameters, and external parameters.

1. Geometric parameters. The dimensions of each component in the model itself and the geometric position relationship between them are reflected by this parameter. The manufacturer provides the main parameters.
2. Mass parameters. The accuracy of the dynamics simulation is directly influenced by the mass of system, centroid and rotational inertia. The mass of the components can be obtained from Adams. Models can be simplified by combining or deleting components.
3. Mechanical parameters. Young's modulus, stiffness, poisson's ratio, etc., are determined according to the material. Alternatively, it can be selected in the material library of Adams.
4. External parameters. These primarily include the working oil pressure of the brake, the amount of pre-pressure, the speed of rotation of the brake disc, etc., according to the operating regulations of the brake for the bridge erecting machine to determine.

The complete brake parameters obtained from the literature^21^ in combination with the measured parameters of the model are shown in Table 1.

**Table 1:** Main parameters of the brake machine.

Basic parameters	Numerical values	Basic parameters	Numerical values
Overall length of the machine (mm)	509	Total weight (kg)	118
Overall height of the machine (mm)	460	Brake operating oil pressure (MPa)	11 ~ 12
Brake disc thickness (mm)	30	Inner diameter of the hydraulic cylinder (mm)	160
Friction piece area (mm^2^)	34,063.884	Piston rod diameter (mm)	70

First, the brake arm, center axis, and axle barrel models were built in SolidWorks and imported them into Adams, set the material parameters of each part, and checked that the centroid coordinates of each component were correct. Table 2 shows the kinematic joints based on the rotation relationships of the individual components.

**Table 2:** Kinematic joints.

Serial number	Name of kinematic subsets	Active objects	Passive objects
1	Revolute joint	Shaft cylinder	Brake arms
2	Fixed joint	Central axis	Shaft cylinder
3	Fixed joint	Base	The ground
4	Fixed joint	Gate tiles	Friction pads
5	Spherical joint	Friction pads	Base

After all kinematic joints were set up, the model was checked for redundant constraints. The friction coefficients between the various components were determined by reference to the contact parameters for steel materials in the machinery manual^22^.

After all the kinematic joints were established, it was necessary to transform the brake arms and center axis into flexible bodies, considering that in the actual motion process, the brake arms and center axis would be deformed during the high clamping force generated by the hydraulic cylinder^23^.

After opening the *igs* format file exported in SolidWorks of the brake arms and center axis in Abaqus, the material parameters were determined as shown in Table 3.

**Table 3:** Material parameters^24^.

Name of material	Density (kg/mm^3^)	Young's modulus (MPa)	Poisson's ratio
Steel	7.85E−9	206,000	0.3

**Figure 3:**
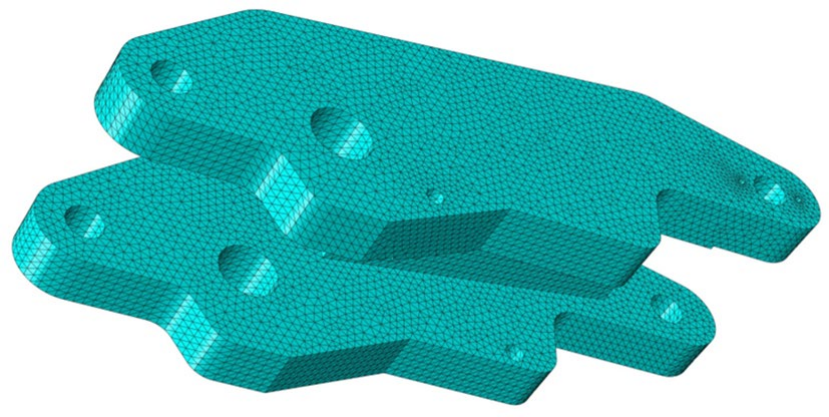
Grid diagram of the brake arm (Abaqus v6.3^26^).

**Figure 4:**
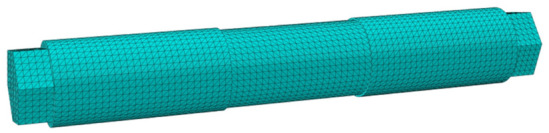
Grid diagram of the central axis (Abaqus v6.3).

After assembly in Abaqus, the mesh was divided, and MPC constraints were set at the location where the part and adjacent parts add kinematic sub-structures as an interface between the flexible body part and the outside environment. The analysis steps were defined as linear perturbation frequency extraction and linear perturbation substructure generation. As shown in Figs. 3 and 4, after meshing the part, the number of nodes in the center axis was 78,295, and the number of cells was 53,064; the number of nodes in the brake arm was 238,628, and the number of cells was 162,148.

The keywords were modified in the JOB interface, then imported the output file into the Adams interface.

- Mass matrix = yes.
*flexible body, type = Adams.
*element recovery matrix,position = averaged at nodes S,E.

After exporting the INP file, the Abaqus command was opened to generate the MNF flexible file. Re-added the kinematic joints of brake arms and center axis in Adams. After the rigid-flexible coupling model was established, the friction coefficients of each kinematic joints and parameters such as simulation time and simulation speed were set, and the motion performance of the mechanism was analysed by post-processing the output data curve^25^.

## Determination of parameters for the simulation of disc springs

**Figure 5:**
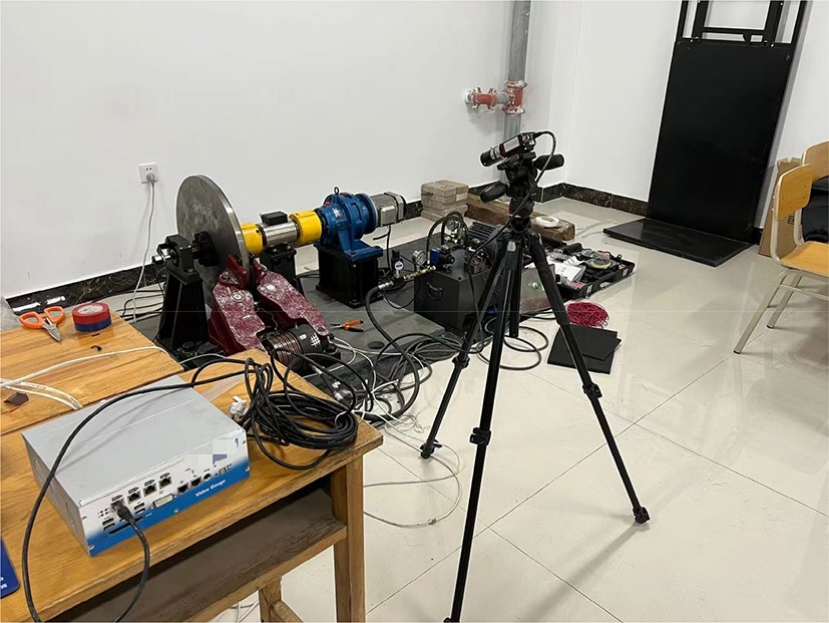
Mechanical properties of disc springs.

Plastic deformation of the disc springs occurs after the brake has been in working condition for a period of time. The original formula for calculating the stiffness is no longer applicable and it is necessary to calibrate the displacement-load curve of the disc spring to measure the current stiffness of disc spring. The disc spring set consists of 11 discs with a total compression of 22.9 mm. To simplify the calculations, the set was reduced to a spring for simulation. As shown in Fig. 5, this experiment was carried out using a non-contact optical instrument to determine the stiffness and pre-pressure values of the disc springs.

**Figure 6:**
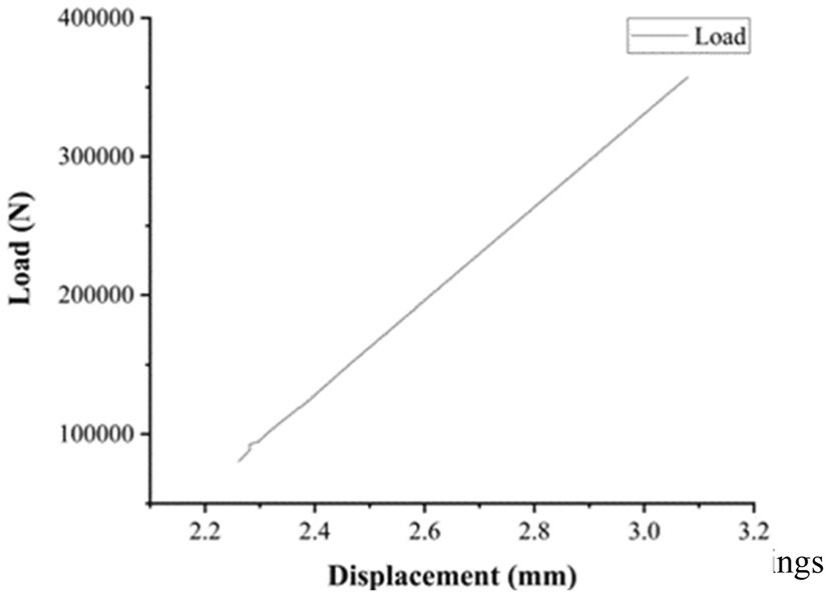
Displacement-load fitting curve for the disc springs.

As shown in Fig. 6, fitting the experimental results proves that the disc spring pre-pressure of 21,565.1176 N and stiffness of 335,952.38018 N/mm.

## Brake mechanical analysis

**Figure 7:**
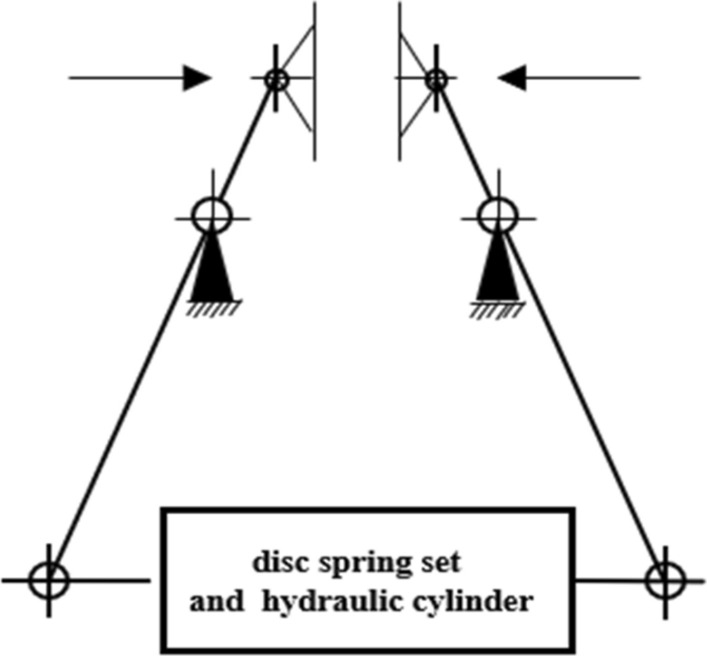
Schematic diagram of the brake forces.

**Figure 8:**
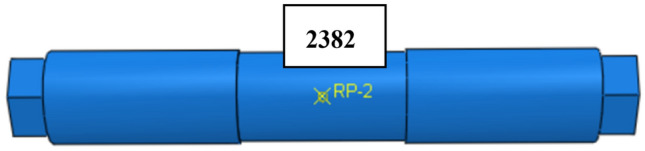
Location of central axis selection points and unit numbering (Abaqus v6.3).

**Figure 9:**
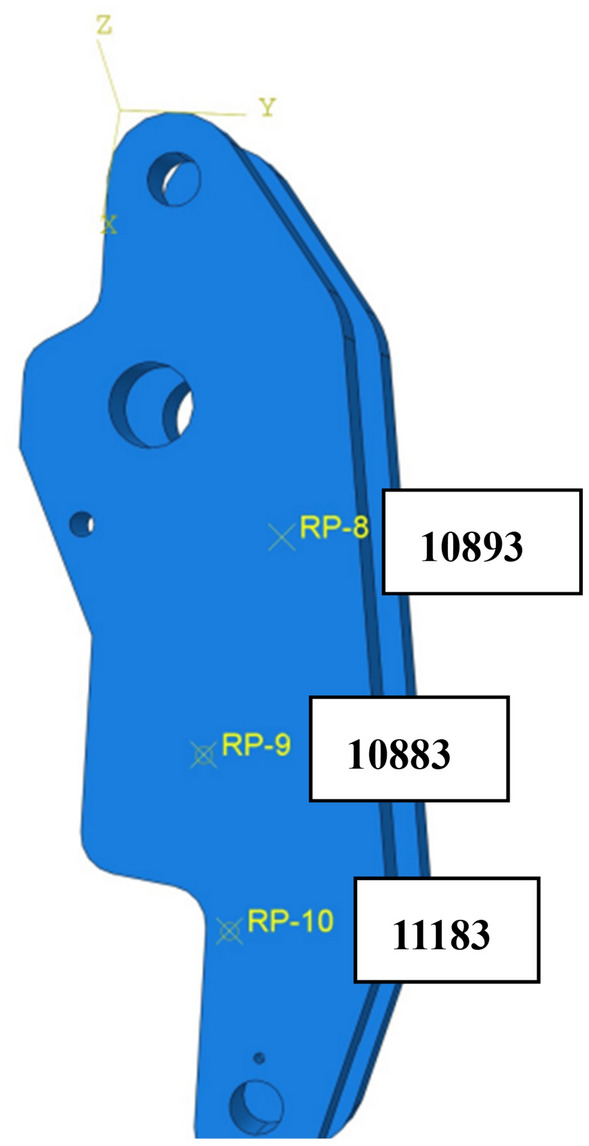
Position of the brake arm selected points and nodes number (Abaqus v6.3).

The *igs* format file of brake model was imported into Abaqus for mechanical analysis. Figure 7 is the schematic diagram of the brake forces. When the brake is closed, the rear part of brake arms are subjected to the resilience of the disc springs, and the front part of brake arms connect the friction pads which clamp the brake rotor. When the brake is released, the brake arms are subjected to the joint action of the disc springs and hydraulic cylinder, causing the friction pads to move away from the brake rotor. Four key-points on the brake arm were selected to extract the stress and strain during the movement to verify the accuracy of the model. As shown in Figs. 8 and 9, the points should be selected to avoid stress concentration points as far as possible to avoid deviations. The midpoint and quarter-points were selected between the center of each rotation axis of the brake arm because their characteristics of data are obvious.

## Simulation test of brake strain

The positions of brake components were calibrated to the realistic state of the brake model, and given that the simulation needs to be consistent with the reality, the load applied during the simulation was the force resulting from the joint action of the hydraulic cylinder and the pre-pressure of disc springs.

15 $$F_{M} = F_{S} + F_{O}$$

where $F_{M}$ represents the simulation of the applied oil pressure, $F_{S}$ is the actual pre-pressure of disc springs, and $F_{O}$ is the actual oil pressure.

## Experimental design

**Figure 10:**
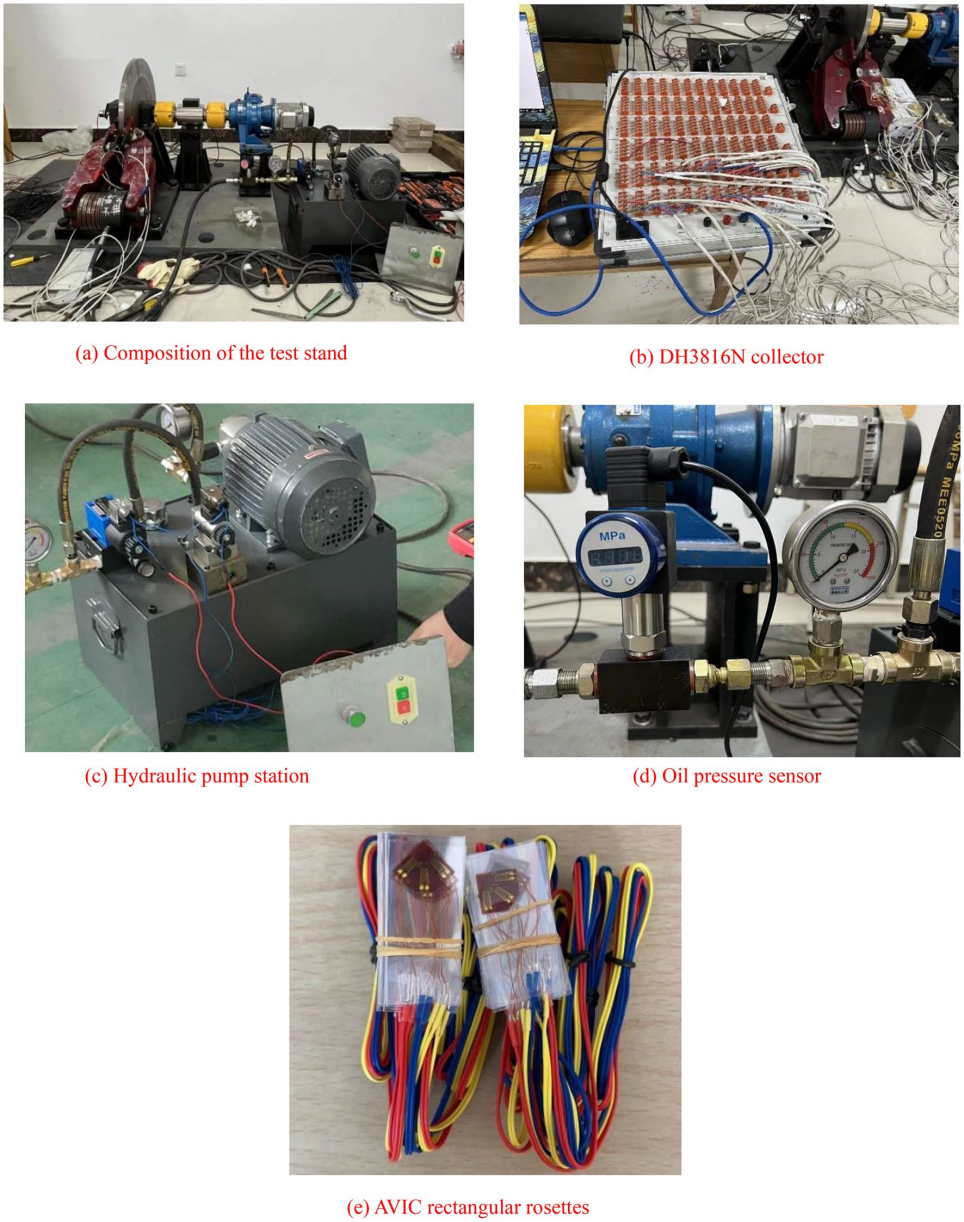
Diagram of experimental equipment.

As shown in Fig. 10, according to the composition of the actual working conditions of the brake, the experiment was designed with an oil pressure pump station, a hydraulic disc brake full scale model, a brake plant, a motor, and an oil pressure sensor. The nodes strain was measured using a rectangular rosette under the oil pressure of 9 MPa. As shown in Fig. 10(b), the experimental data were gathered using the DH3816N collector.

The inverse of the volume compression coefficient of the liquid, known as the bulk modulus of elasticity of the liquid, is expressed as β. The bulk modulus of elasticity of the hydraulic fluid is related to the temperature, pressure and the air contained in the fluid. In this paper, the bulk modulus of elasticity of the hydraulic fluid is 1000 MPa. The temperature of oil pressure is controlled at 30–50 °C.

The clearance of the brake shoe of the model was adjusted so that it did not generate pressure, and the oil pressure of the pump station was determined to be 9 MPa by the indication of the oil pressure sensor. The rectangular rosettes were pasted and the availability of it was tested by the multimeter. After completing the test without errors, the rectangular rosettes were connected to the collector. After the indicator of the strain collector was stable, the data of each point within the test time of 80–120 s were selected to calculate the average value. A total of three experiments were repeated, and the mean values of the strain obtained from the experiment were compared with the strain of simulation model under the same motion process.

Fig. 10(e) is the rectangular rosette. The principle of calculation is as follows.^27–29^:

16 $$\varepsilon_{x} = \varepsilon_{0^\circ }$$

17 $$\varepsilon_{y} = \varepsilon_{90^\circ }$$

18 $$\gamma _{{xy}} = \varepsilon _{{0^{^\circ } }} + \varepsilon _{{90^{^\circ } }} - 2\varepsilon _{{45^{^\circ } }}$$

19 $$\tan 2\alpha_{0} = - \frac{{\gamma_{xy} }}{{\varepsilon_{x} - \varepsilon_{y} }}$$

20 $$\left. {\begin{array}{*{20}c} {\varepsilon_{max} } \\ {\varepsilon_{min} } \\ \end{array} } \right\} = \frac{{\varepsilon_{x} + \varepsilon_{y} }}{2} \pm \sqrt {\left( {\frac{{\varepsilon_{x} - \varepsilon_{y} }}{2}} \right)^{2} + \left( {\frac{{\gamma_{xy} }}{2}} \right)^{2} }$$

The strain measurement technology of resistance has become a fairly mature test method and is widely used in the field of engineering technology^30^. The test procedure is as follows^31^.

1. The rectangular rosettes are applied to each measurement point, and the insulated wires are soldered to each of the rectangular rosettes.
2. The resistance of the three strain gages on the rectangular rosette is measured using a multimeter to ensure that the rectangular rosette is usable.
3. The wires are connected to the different pathways of the DH3816N collector.
4. When the data displayed on the collector are balanced, the experiments of brake operation will be repeated for three times.
5. Export the experimental data.

## Experimental verification

As shown in Table 4, the experimental results show that the maximum and minimum strains at points 11,183, 10,883, and 10,893 on the brake arm are all within the 15% error range. The maximum strain at point 2382 on the axis is not within the measurement range of the collector for strain and is not comparable; the minimum strain is within the error range. The kinetic simulation model is reasonable and accurate. The model can be used for extracting faults of the brake, loading the load spectrum during the operation of the bridge erecting machine and diagnosing faults of the brake. The model is essential for predicting the remaining life and diagnosing faults of the brake.

**Table 4:** Comparisons between simulation results and test data.

Location of measurement points	Node number	Maximum strain $\sigma_{1}$($\mu \varepsilon$ )	Simulated maximum strain value ($\varepsilon$ )	Relative error (%)	Minimum strain $\sigma_{2} (\mu \varepsilon)$	Simulated minimum strain value ($\varepsilon$ )	Relative error (%)
Brake arms	10,883	397.0153	3.7646E−04	5.4	− 502.3893	− 4.9252E−04	1.97
	11,183	46.3151	3.9949E−5	13.74	− 14.6713	− 1.2797E−05	12.77
	10,893	451.135	4.30E−04	4.64	− 151.402	− 1.36E−04	9.882
Center axis	2382	0.47002	1.0888E-07	–	− 23.88998	− 2.1736E-05	8.3

## Simulation of performance degradation and extraction of fault features

When a braking system is in service for an extended period, the performance of components will degrade and impact the working condition of braking system^32^. Large lifting machinery requires long-term braking when walking on rough roads, and the vibration has the potential to cause fatigue cracking of the brake. The friction coefficients and damping system between components greatly influence the vibration of brake^33^. In this paper, the faults of the prolonged operation on braking system performance were simulated in the form of reduction of disc springs stiffness and insufficient oil pressure. In practice, when the faults occur, it will have a huge impact on the safety of the braking system. It will cause problems such as insufficient braking system pressure or wear of the brake shoes. Currently, the method to solve the similar problems mainly relies on workers climbing to the brake of the bridge erecting machine for maintenance. The simulation model was used to simulate the occurrence of the faults to extract the fault characteristics. It provided the basis for the judgment of the cause of the faults, and it also proved that it was feasible to use the simulation model for fault analysis.

### Reduced stiffness of disc spring

**Figure 11:**
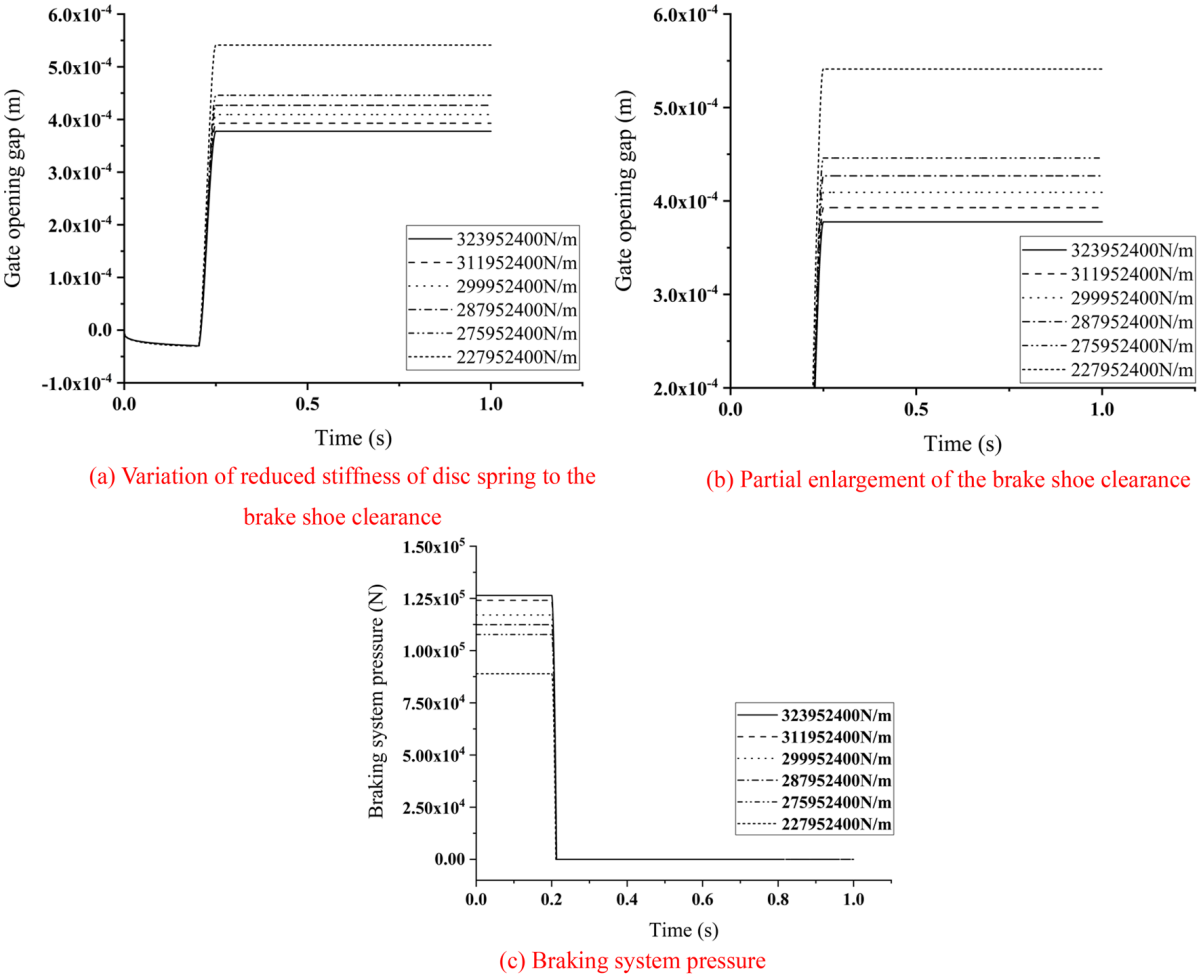
Variation of braking system parameters with reduction of stiffness of disc spring.

The simulation model was adjusted to simulate the condition of the brake releasing. The oil pressure was set at 11.5 MPa, the brake was released in 0.2–0.25 s, and the compression length of the disc springs was 22.9 mm. The reduction of stiffness of disc spring also resulted in a decrease in disc springs pre-pressure. Actuating the brake rotor by applying torque in Adams. The clearance of the brake pad and the braking system pressure were extracted. As shown in Fig. 11.

According to the provisions of the brake design parameters, the brake shoe retraction distance should be 0.7–1 mm, and the distance of one-side retraction should be 0.35–0.5 mm. It could be seen that in the process of the reduction of the stiffness of disc spring, the change of brake shoe clearance did not exceed this limit at first, but as the stiffness continued to reduce, the closing time also increased. As shown in Fig. 11(c), when the brake shoe clearance was between 0.35 mm and 0.5 mm, the pressure of the braking system was continuously decreasing, which proved that the performance of the braking system was gradually degraded. When the brake shoe clearance was beyond 0.5 mm, it proved that the braking system was failed.

### Insufficient oil pressure

**Figure 12:**
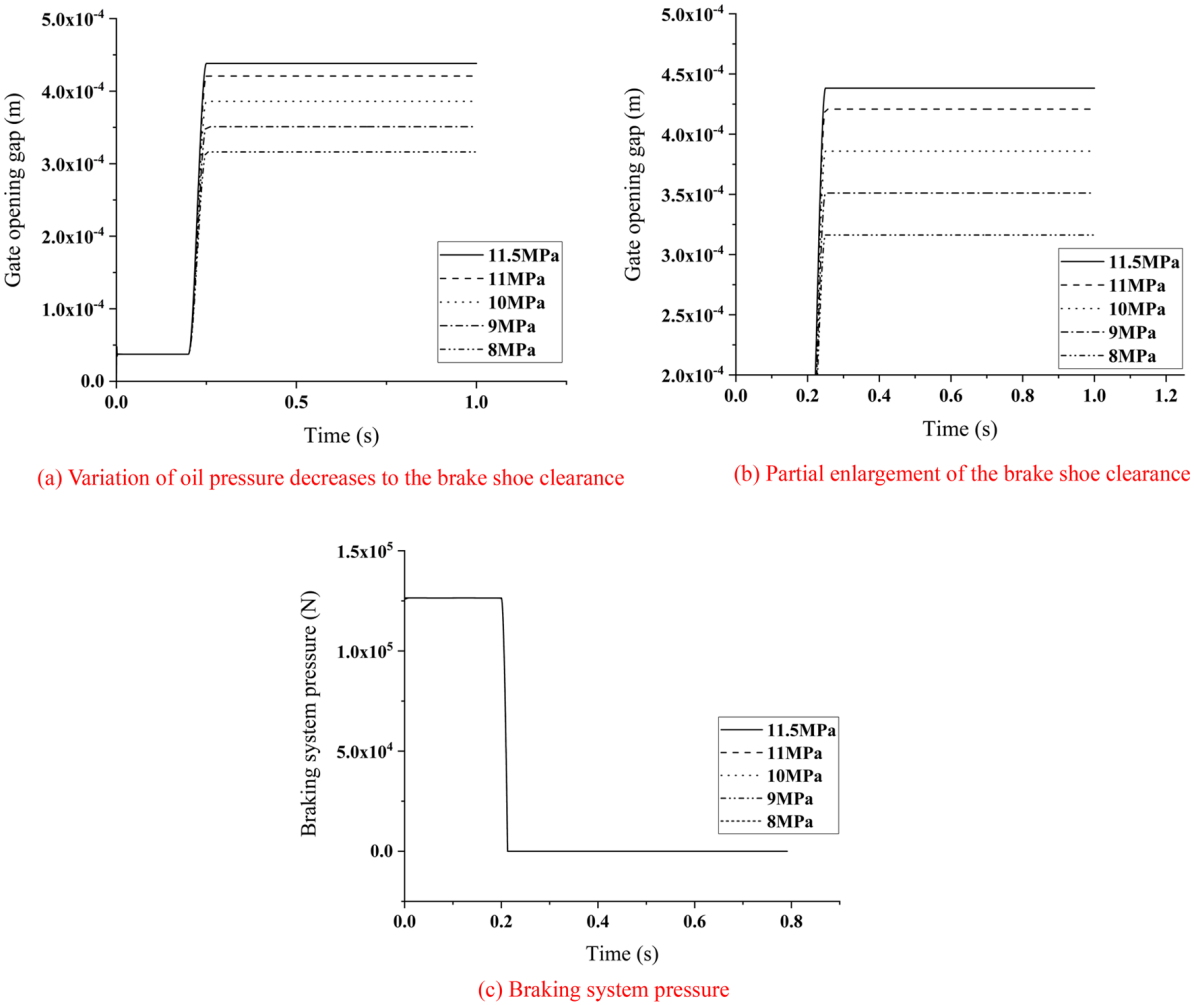
Variation of braking system parameters with reduction of oil pressure.

The condition that the braking system had been working for a period of time under the oil pressure of 11.5 MPa was simulated. The hydraulic station faulted because the hydraulic system was not tightly sealed, which caused the oil pressure was insufficient. The variation of brake shoe clearance and the variation of braking system pressure were extracted under different oil pressures, as shown in Fig. 12.

According to Fig. 12, it could be seen that when the oil pressure decreased, the brake shoe clearance was still within the specified range of 0.35–0.5 mm but the pressure of the braking system did not change. At this time, it caused the performance degradation of braking system, but it was not beyond the safe range. With the further reduction of oil pressure, the brake shoe clearance was less than 0.35 mm. At this time, the braking system was disabled because the brake jammed.

## Conclusion and prospect

This paper uses the multi-body dynamics modeling method to simulate and analyse the working condition of the brake used in the bridge erecting machine. Considering the actual state that the disc springs will produce plastic deformation, the experiment of the displacement-load curve of the disc springs is carried out. The actual test verified the accuracy of the simulation model. The possible fault characteristics of reduction of stiffness of disc spring and insufficient oil pressure of the brake are simulated by Adams. According to the results of variation of brake shoe clearance and the variation of braking system pressure, two causes of faults can be accurately distinguished. It is concluded that the first stage do not affect the normal operation of the brake when the fault characteristics appear. When the fault characteristics are beyond the critical threshold, the faults of brake have occurred. Obviously, the occurrence of the reduction of stiffness of disc spring is characterized by increased clearance of brake shoe and decreased pressure of braking system. The fault of insufficient oil pressure is characterized by decreased brake shoe clearance and stable pressure of braking system. When the faults occur, it will impact its safety performance. Evidently, the results prove that it is very feasible to extract fault characteristics through simulation model to assist in determining the cause of faults.

Currently, the research focused on the process of establishment and the analysis of usability of the simulation model of hydraulic disc brakes without considering the friction and the influence of the hydraulic system on the brake. The follow-up research can be further improved for heat-engine coupling or hydraulic control. The method of modeling analysis of brakes on hoisting system used in this paper can significantly reduces the costs of maintenance, and has a great reference significance for large-scale mechanical equipment such as flatcar hoist and mine hoist, of which has large volume, complex working environment, and great importance. Based on this, Internet technology can be used to establish an intelligent integrated monitoring platform to solve the technical key problems of braking system of large-scale equipments. At the same time, it can improve the safety theory system and form the independent real-time warning system to reduce the potential danger of major accidents in the braking system, to ensure the operation of hoisting machinery stable and reliable.

## Data Availability

The data supporting this study's findings are available from the corresponding author upon reasonable request.

## Abbreviations

PCNN: Pulse Coupled Neural Network
MPC: Multi-point Constraints
MNF: Modal Neutral File
JOB: Abaqus analysis tasks
INP: The inp, the file is the abbreviation of the input
